# Propene Polymerization with *C*_1_-Symmetric Fluorenyl-Metallocene Catalysts

**DOI:** 10.3390/polym9110581

**Published:** 2017-11-06

**Authors:** Laura Boggioni, Massimiliano Cornelio, Simona Losio, Abbas Razavi, Incoronata Tritto

**Affiliations:** 1 CNR Istituto per lo Studio delle Macromolecole (ISMAC), Via E. Bassini, 15-20133 Milano, Italy; boggioni@ismac.cnr.it (L.B.); cornelio@ismac.cnr.it (M.C.); s.losio@ismac.cnr.it (S.L.); 2 Total Petrochemicals Research, Zone Industrielle C, B-7181 Feluy, Belgium; abbasrazavi@synet.be

**Keywords:** propene, metallocene catalysts, microstructure

## Abstract

Propene homopolymers have been produced by employing three *C*_1_-symmetric metallocene molecules (**1**, **2** and **3**), each having *t*-butyl substituent(s) on the *C*p, on the fluorenyl or on both aromatic moieties activated with methylaluminoxane at different polymerization temperatures and monomer concentrations. Polymers’ microstructures determined by ^13^C NMR spectroscopy suggest that the otherwise dominant alternating mechanism governed by the chain migratory insertion is largely replaced by the competing site epimerization mechanism, as a direct result of the imposing steric bulk of the *t*-butyl substituent on one of the distal positions of the *C*p moiety. This phenomenon is more pronounced with **3** when a second *t*-butyl is present in the same half-space of the molecule making the site epimerization mandatory. The lower activity of catalyst **3** with respect to catalyst **2** is also in line with the necessity for the polymer chain to back-skip (or the site to epimerize) to its original position before the subsequent monomer insertion. Chain end group analyses by ^1^H NMR spectroscopy have revealed that the formation of vinylidene end groups, either via β-H elimination or as a result of direct chain transfer to the monomer after an ordinary 1,2-insertion is the prevailing chain termination route. A correlation between the relative concentrations of vinylidene end groups of polypropene (PP) polymers produced with each catalyst and the corresponding polypropenes’ molar masses was found, indicating the lower the relative concentrations of vinylidene end groups, the higher the molar masses.

## 1. Introduction

The advent of homogeneous *ansa* metallocene catalysts for stereospecific olefin polymerization had been considered a groundbreaking event in this industrially very important process [1,2,3]. The possibility of rationally modifying the ligand structure of these systems allow one to have a high degree of control over polymer tacticity, comonomer incorporation/distribution, molar masses and molar mass distributions, thus over their physical and mechanical properties. The development of *C*_s_-symmetric fluorenyl-containing metallocenes of the type Me_2_C–(C_5_H_4_)(C_13_H_8_)MCl_2_ (e.g., M = Zr) [4,5,6,7,8,9], activated with methylaluminoxane (MAO), offered the possibility of synthesizing highly crystalline syndiotactic polypropene (sPP). Since the discovery of these catalyst systems, their catalytic behavior and the origin of their syndiospecificity have been the subject of intensive debate by many authors in numerous publications [8,9,10,11,12,13,14,15]. These systems endowed with two enantiomorphic sites offer the possibility of testing the existing theories put forward on the origin of the catalyst’s stereocontrol [16,17].

**Figure 1:**
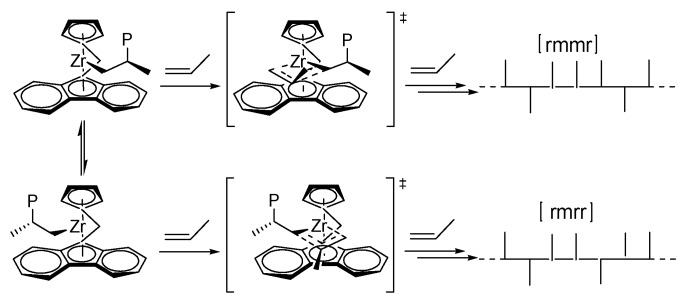
Errors in syndiotactic polypropene with *C*_s_-symmetric metallocenes: isolated stereoerrors (rmmr) by site control and (rmrr) by site epimerization.

The current views on syndiospecificity could be summarized as the following: (i) *C*_s_-symmetric metallocene molecule possesses one bulky substituent (fluorenyl), which directs the growing polymer chain toward the less hindered cyclopentadienyl side; (ii) the fluorenyl possesses an empty space in the central region wide enough to accommodate the methyl group of propene coordinated in trans arrangement with respect to the polymer chain; and (iii) the incoming monomer and the growing polymer chain alternate between the two enantiotopic sites, after each monomer insertion following a migratory insertion (Figure 1). The presence of stereoerrors such as *rmmr* is consistent with enantiomorphic site control mechanism, whereas the presence of a variable amount of *rmrr* is indicative of occasional skipped insertions process.

The typical stereoerrors that decrease the syndiotacticity of sPP are due to monomer enantiofacial misinsertions, leading to *rmmr* sequences and site epimerization related errors (back skip of the polymer chain) leading to an isolated *m* unit in the backbone of the chain and thus to *rmrr* sequences. Syndiotactic polypropeneis are thermoplastic material with a high melting point and high crystallinity, both influenced by the number of stereoerrors.

After the initial reports, several authors developed bridge-modified analogs, including doubly bridged metallocenes, a number of fluorenyl substituted metallocenes [13,14], as well as cyclopentadienyl-substituted variants of the parent metallocene which lead to PP chains with different microstructures and thus with different melt transition temperatures, e.g., incorporation of a substituent at the 3 position of the cyclopentadienyl ring induces desymmetrization of the metallocene to *C*_1_ symmetry [18,19]. Consequently, the tacticity of propene homopolymers that are produced by using *C*_1_-symmetric catalysts is no longer of syndio type. The polymer microstructure now greatly depends on the nature/size of the substituent on the *C*p group and the degree of steric hindrance it causes. When the substituent is a methyl or an *i*-propyl group, a hemi-isotactic or atactic polypropene is obtained; however, when the substituent is the bulkier *t*-butyl or tri-methylsilyl, a predominantly isotactic polypropene is formed. The mechanism which leads to the formation of isotactic polypropene with *C*_1_-symmetric Me_2_C–(3–*t*–Bu–C_5_H_3_)(C_13_H_8_)MCl_2_/MAO catalyst has been a subject of debate. To confirm or discard one or the other proposed mechanism, a number of *C*_1_-symmetric metallocene molecules have been synthesized and studied more recently [20].

We report herein on the study of the propene polymerization catalysis with *C*_1_-symmetric cyclopentadienyl-fluorenyl-containing metallocenes bearing one or two *t*-butyl substituents at the 3 position of *C*p or at 3 position of the fluorenyl group. The main objective of this investigation was to assess the impact of these substitutional changes both on the catalytic performances and on polypropene microstructures and their molar masses under conditions close to those used in industrial processes. Thus, three *C*_1_-symmetric metallocenes were subjected to MAO-cocatalyzed propene homopolymerizations at different temperatures and monomer concentrations. The tacticities and stereoerrors of the polymers were investigated by ^13^C NMR analysis, the best tool for elucidating polymer stereochemistry and the polymerization mechanism. A comparison of the polymer microstructures on the basis of statistical analysis was performed, and mechanisms that lead to the observed microstructures were tested. Results were compared to those found with the reference Me_2_C–(3–*t*–Bu–C_5_H_3_)(C_13_H_8_)ZrCl_2_ (**4**) and C_s_-symmetric Me_2_C–(C_5_H_4_)(C_13_H_8_)ZrCl_2_ (**5**) [4,5,6,7,8,9,10,11,12,13] catalysts. Finally, the nature and concentration of the chain end groups determined by ^1^H NMR analysis were correlated to the molar masses of the polymers.

## 2. Materials and Methods

All experiments were performed under nitrogen atmosphere in the glove box or using standard Schlenk techniques. Complexes were provided by Total, Feluy, Belgium. Methylaluminoxane (MAO) (10 wt % as toluene solution, Crompton) was dried (50 °C, 3 h, 0.1 mm Hg) before use. Nitrogen and propene gases were dried and deoxygenated by passage over columns of CaCl_2_, molecular sieves, and BTS catalysts. Mixed metal catalysts were composed of CuO and ZnO from Aldrich, Milan, Italy. Toluene was distilled from sodium under nitrogen atmosphere. C_2_D_2_Cl_4_ was purchased from Cambridge Isotope Laboratories Tewksbury, MA, USA and used as received.

All homopolymerizations were carried out in a 250 mL laboratory stainless steel autoclave Buchi, Uster, Swisse, equipped with an external thermostatic bath, for temperature regulation, connected to a Temperature Control Unit Polystat, cc2 model, Huber, Offernbuy, Germany. The internal reactor temperature was monitored with a thermocouple (Pt100 model, Achelit, Rho, Italy) inserted into the reactor vessel and connected to the Temperature Control Unit. The reactor was equipped with a helicoidal stirrer, and was connected to high-pressure gases and a high-vacuum system throughout standard Swagelok’s instrumentation, Solon, OH, USA, including compression fittings, valves, tubing and gauges. The manipulation of air sensitive catalysts and co-catalysts were carried out in a flame or oven-stored Shlenk type glassware, Valchimica, Milan, Italy, using high vacuum line and glove-box techniques. The solvent was freshly distilled on sodium and stored on molecular sieves before every use.

*General polymerization procedure*. A typical propene polymerization experiment was carried out in a 250 mL stainless steel autoclave, under slurry experimental conditions. Before starting the polymerization reactions, the reactor was evacuated for 60 min at 90 °C and conditioned three times with nitrogen. In the meantime, two Schlenk tubes were charged with the right amount of metallocene precursors and *d*-MAO dissolved in toluene, respectively. After cooling down to reaction temperature, the reactor was filled with the measured amount of toluene and with the solution of *d*-MAO in toluene, that was prepared in advance. A total of 50 mL toluene solvent was used; Co-catalyst: *d*-MAO, dissolved in 3 mL of toluene; catalyst loading: 5 μmol of metallocene dissolved in 2 mL of toluene; Al/Zr: 3000 m.r. Finally, a weighted amount of propene (ranging from 25 to 30 g) was transferred into the reactor. After thermal equilibration, the polymerization reaction was initiated by injection of the solutions of the corresponding metallocene precursors via syringe. The polymerizations were quenched by addition of 2 mL of ethanol, and the polymers were precipitated in ethanol. The products were filtered, re-dissolved in boiling toluene and re-precipitated in ethanol under stirring. The products were filtered and dried under vacuum at 70 °C overnight. Propene concentration in toluene was calculated according to Henry’s law:

(1) $$\text{Henry}\;\text{law}=C_{\mathrm{Propene}}=P_{\mathrm{Propene}}\times H_{o}\exp\left(\frac{\Delta H_{L}}{RT}\right)$$

*C*_Propene_ = propene concentration (mol/L); *P*_Propene_ = propene pressure (atm); *H_o_* = Henry coefficient = 0.00175 mol·L^−1^·atm^−1^; Δ*H*_L_ = enthalpy of solvation of propene in toluene = 3295.6 cal·mol^−1^; *R* = 1.989 cal·mol^−1^·*K*^−1^.

*Polymer characterization*. NMR spectra were recorded on a Bruker NMR Avance 400 Spectrometer operating at 400 MHz (^1^H) and 100.58 MHz (^13^C) working in the pulse Fourier transform mode at 103 °C. ^1^H and ^13^C experiments were performed with a 10 mm probe, in C_2_D_2_Cl_4,_ and referred to hexamethyldisiloxane (HMDS). Molar masses and molar mass distributions (*M*_w_/*M*_n_) were determined in *o*-dichlorobenzene at 145 °C by a GPCV2000 high temperature size exclusion chromatography (SEC) system from Waters, Milan, Italy, equipped with two online detectors: a viscometer (DV) and a differential refractometer (DRI). The column set was composed of three mixed TSK-Gel GMHXL-XT columns from Tosohaas, Rivoli, Italy. The universal calibration was constructed from 18 narrow *M*_w_/*M*_n_ polystyrene standards, with the molar mass ranging from 162 to 5.48 × 10^6^ g·mol^−1^.

## 3. Results and Discussion

### 3.1. Polymer Synthesis and Analysis

**Scheme 1:**
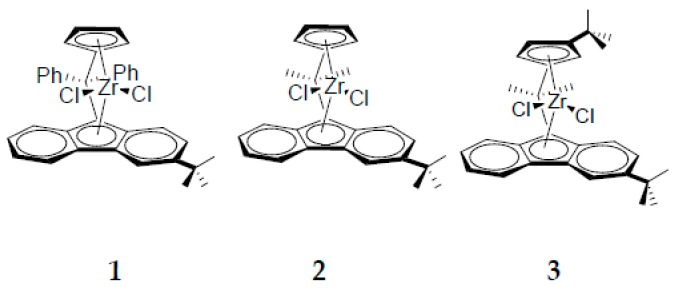
The *C*_1_-symmetric catalysts (**1**–**3**) used in propene homopolymerizations.

The three metallocene precatalysts (**1**, **2** and **3**) shown in Scheme 1 were activated with MAO and evaluated in homogeneous polymerization of propene (in autoclave, about 25 g of propene, Al/Zr molar ratio of 3000 in a total 50 mL of toluene, at three polymerization temperatures, 30, 50 and 70 °C). In Table 1, selected results of synthesis and molar masses of polymers obtained from catalysts **1**, **2**, and **3** are listed.

The diphenyl bridged catalyst **1** with *t*-butyl substituent at the 3 position of fluorenyl is 2–3 fold more reactive than the analogous isopropylidene-bridged catalyst **2**, in line with results reported for other substituted metallocenes with a similar difference in the bridge, and thus is the most active. On the other hand, comparison between catalytic performances of **2** and **3** reveals that the *t*-butyl group on the distal position of the *C*p moiety, with its steric bulk closer to the metal center, leads to the lowest activity for catalyst **3**. Moreover, the polymerization temperature influences the activities in an unusual way and contrary to what is generally observed in olefin polymerization with these types of catalyst. In these three cases, the higher the polymerization temperature the lower the observed activity for all the catalysts. This may lead one to conclude that the phenomenon may be related to the lower stability of the catalyst at higher temperatures.

SEC analysis of the polymers obtained showed that catalyst structure influences molar masses (*M*_w_). The diphenyl–bridged catalyst system (**1**), the most active one, yields the highest molar masses, while by comparing the isopropyl-bridged catalysts **2** and **3**, the latter system, the least active one, yields molar masses greater than **2**. In addition, polymer’s *M*_w_ depends on the polymerization temperature, where the higher the temperature, the lower the *M*_w_, as expected due to an increase of chain transfer processes and β-hydride elimination reactions with increasing in temperature. In general, molar mass distributions are around two, even at higher polymerization temperatures, consistent with single site nature of the catalysis.

### 3.2. Polypropene Microstructure

**Figure 2:**
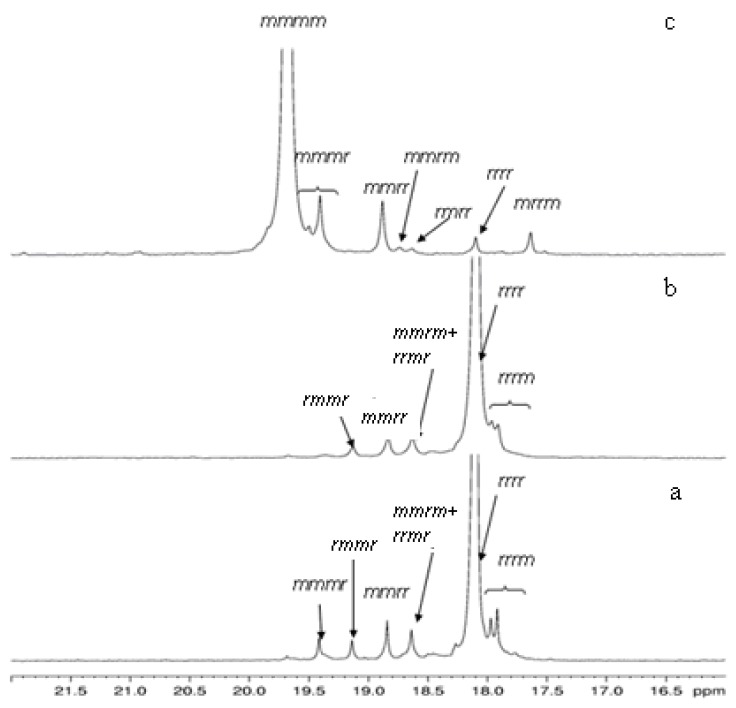
Methyl region of ^13^C NMR spectra (108.58 MHz, C_2_D_2_Cl_4_, 103 °C) of polyPP samples prepared at 50 °C by: catalyst **1** (**a**); catalyst **2** at (Propene) = 13.9 M (**b**); and catalyst **3** (**c**).

The microstructures of all the homopolymers obtained with the three above mentioned catalyst systems were investigated by ^13^C NMR spectroscopy. In Figure 2, the methyl region of ^13^C NMR spectra of PP obtained from the three catalysts are displayed. The spectra show the resonances indicative of the presence of the following typical pentads: *mmmm* (19.60 ppm), *mmmr* (19.40–19.31 ppm), *rmmr* (18.96–18.85 ppm), *mmrr* (18.76–18.70 ppm), *rmrr* + *mrmm* (18.65–18.57 ppm), *rrrr* (18.19–18.08 ppm), and *rrrm* (17.99–17.87 ppm). The percentage of syndiotactic (*rrrr*), isotactic (*mmmm*) pentads, and those of two of the most common stereo errors (*rmrr* and *rmmr*) for the homopolymers obtained with the three catalysts are listed in Table 2.

It is evident from the ^13^C NMR spectrum shown above that catalyst **1** produces mainly syndiotactic polypropene (Figure 2a), with the *rmrr* pentad linked to the site epimerization being particularly high, and a tacticity which is lower compared to the tacticities of sPP prepared with analogous *C*_s_-symmetric catalyst under similar conditions [3,4]. The syndiotactic pentad percentage decreases with increasing polymerization temperature; the monomer concentration also seems, as expected, to have an influence on tacticity. The isopropyl-bridged catalyst **2** gives highly syndiotactic polypropene; the *rrrr* and *rmrr* pentads being respectively higher and smaller than those obtained with diphenyl bridged catalyst **1**. The *rrrr* percentage decreases with the increase of polymerization temperature in a way which is similar to the analogous syndiospecific catalyst **1**. The changes in the microstructure of polymers obtained at 50 °C at different monomer concentrations reveals that, by increasing the monomer concentration to 17.7 M, the syndiotacticity decreases. On the contrary, catalyst **3**, which has *t*-butyl substituents at the 3 position of both *C*p and fluorenyl groups, gives highly isotactic polypropene, and both the monomer concentration and the polymerization temperature have a small influence on the polymer microstructure. Thus, interestingly, the polypropene obtained is highly isotactic at 70 °C also.

Detailed analysis of the stereochemistry of poly-α-olefins has been proven to provide new insights into the mechanistic details of catalytic olefin polymerization [21,22,23]. Thus, to gain insights into the origin of the variation of microstructure, the observed polymer tacticity was compared to that predicted by three statistical models utilized by Bercaw for testing mechanism of formation of isotactic polypropene with *C*_1_-symmetric metallocenes [20]. The first is enantiomorphic site control which employs a single site with enantioselectivity α, as predicted by site epimerization. The second is an alternating model that is applicable to a catalyst that regularly alternates insertions between a perfectly stereoselective site (α = 1) and a site having a variable stereoselectivity (β). The third is an alternating model appropriate for a catalyst that regularly alternates insertions between two sites of variable stereoselectivity (α and β). Both alternating models assume that no site epimerization is occurring.

**Figure 3:**
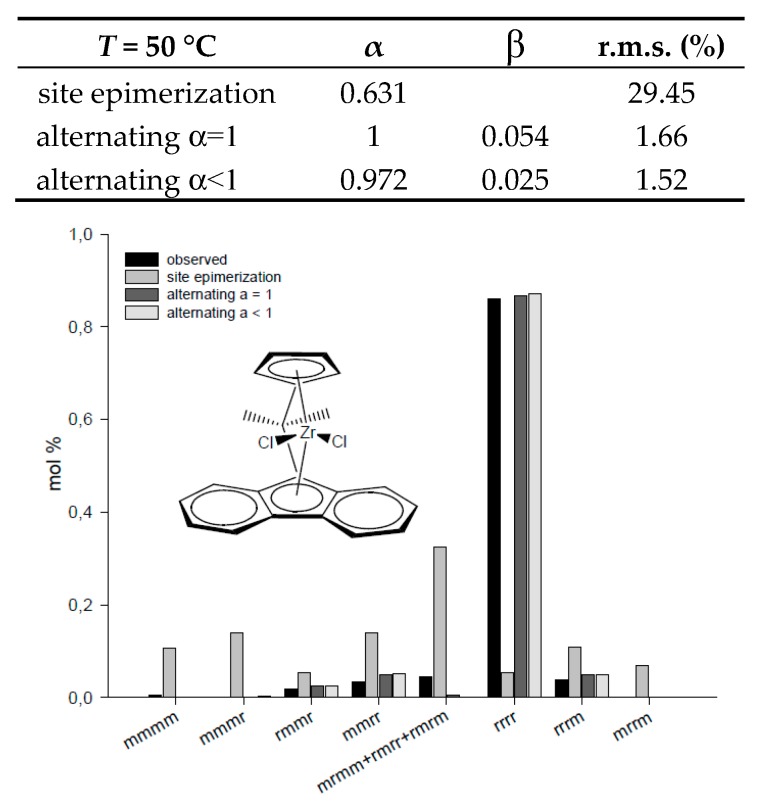
Statistical pentad analysis of a polypropene sample prepared with **5**/MAO at 50 °C.

**Figure 4:**
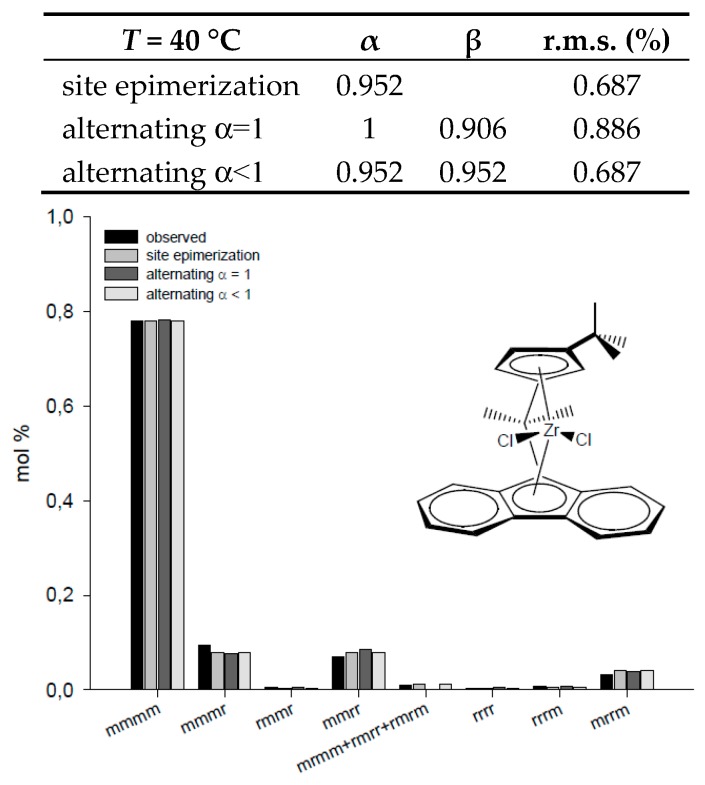
Statistical pentad analysis of a polypropene sample prepared with **4**/MAO at 40 °C [4,20].

The polymer tacticities predicted on the basis of statistical analysis for the polypropene obtained by catalysts **1**, **2** and **3** were calculated and compared to the experimental tacticity. The same analysis for polypropene obtained with *C*_1_-symmetric catalyst **4 [4]**, as applied by Bercaw [20], and with *C*_s_-symmetric catalyst **5**, prepared in our laboratory, are reported for comparison (Table 3, Figure 3 and Figure 4). It is clear that, for the *C*_s_ symmetric catalyst **5**, the root mean square (r.m.s.) errors provided by the fits from the site epimerization mechanism and the alternating one are very different, indicating that the alternating two site mechanism better predicts the syndiotactic microstructure of polypropene produced by this catalyst. Of the two alternating mechanisms the one with two sites of variable stereoselectivity gives the lowest error. On the contrary, for catalyst **4**, which has one *t*-butyl on the *C*p, the r.m.s. errors provided for the three predictive mechanisms are quite similar. Such similarity was considered by Bercaw et al. too close to draw definitive conclusions as to whether the catalytic site epimerization or chain alternating behavior would be the better model to predict the actual microstructure of the polypropene produced. Here, we would like to point out that the lowest r.m.s. errors are found for the site epimerization and for the alternating mechanism with two sites of variable stereoselectivity. In addition, the site epimerization model gives an α value of 0.952; exactly the same α and β values are found for the alternating mechanism with variable stereoselectivity.

**Figure 5:**
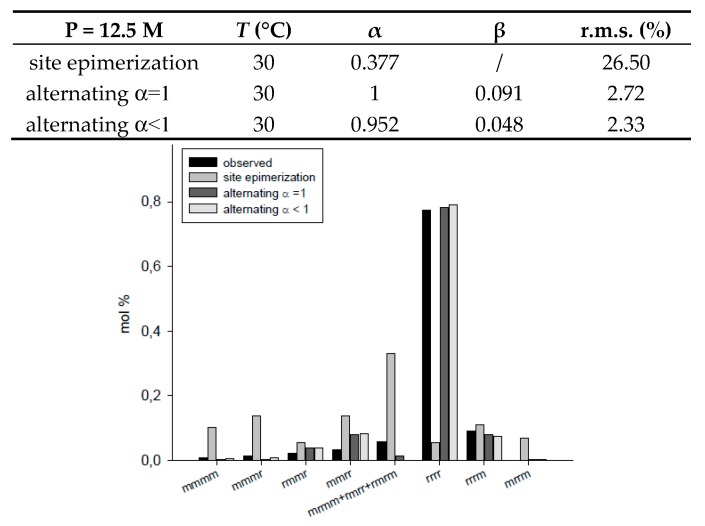

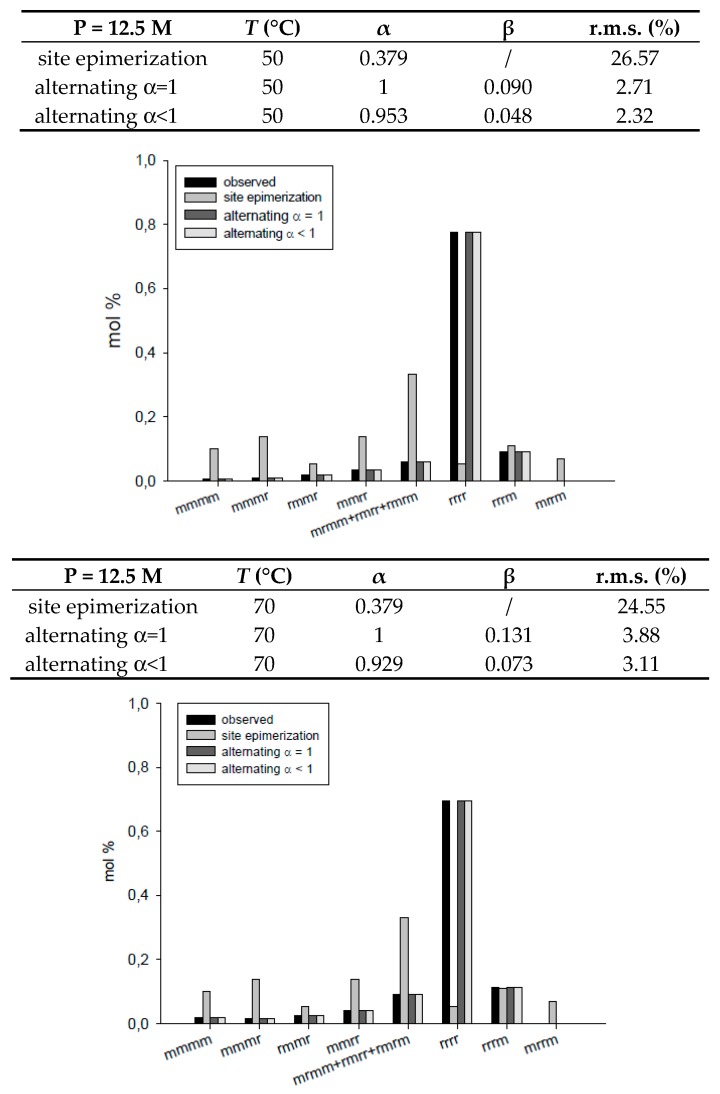
Statistical pentad analysis of a polypropene sample prepared with **2**/MAO at different temperatures.

Now it is of interest to analyze the microstructure of polypropene obtained by catalysts **1**, **2** and **3** at different temperatures and by catalyst **2** at different monomer concentrations at the same temperature of 50 °C (Table 4 and Figure 5).

Regarding catalyst **2**, with one *t*-butyl on the fluorenyl group, results are rather similar to those of catalyst **5** without any substituent, both giving mainly syndiotactic polypropene. Indeed, for all three polymerization temperatures, the epimerization mechanism could not predict the pentad distribution, while both alternating mechanisms well describe the observed microstructure. What seems to be of interest is that errors with site epimerization mechanism for catalyst **2** are lower than for **5**; the opposite holds for the alternating mechanisms. Thus, these results indicate that steric desymmetrization due to *t*-butyl at 3 position of the fluorenyl catalyst is slight. Another interesting observation is that, by raising the temperature the r.m.s. errors for the site, epimerization mechanism decreases slightly, while those for the alternating mechanism increase slightly. The same trend is evident comparing results obtained at two different propene concentrations at 50 °C.

Statistical analysis of polypropene by catalyst 1 reveals results similar to those from 2 (Table 5). Again, by comparing results of catalyst 1 with those obtained with catalyst 5, it is worth noting that errors with site epimerization mechanism for catalyst 1 are lower than for 5; and slightly lower than for 2. The opposite holds for the alternating mechanisms. The steric desymmetrization due to *t*-butyl at 3 position of the fluorenyl of catalyst 1 is greater than in 2, rendering the alternating mechanism to be slightly less favorable and the epimerization moderately more favorable than in 1. Results at 30 and 70 °C confirm that by raising the temperature the r.m.s. errors for the site epimerization mechanism decrease slightly while those for the alternating mechanism increase little; the variations at 50 °C are greater than in the polymers obtained at 70 °C since the propene concentration is greater.

**Figure 6:**
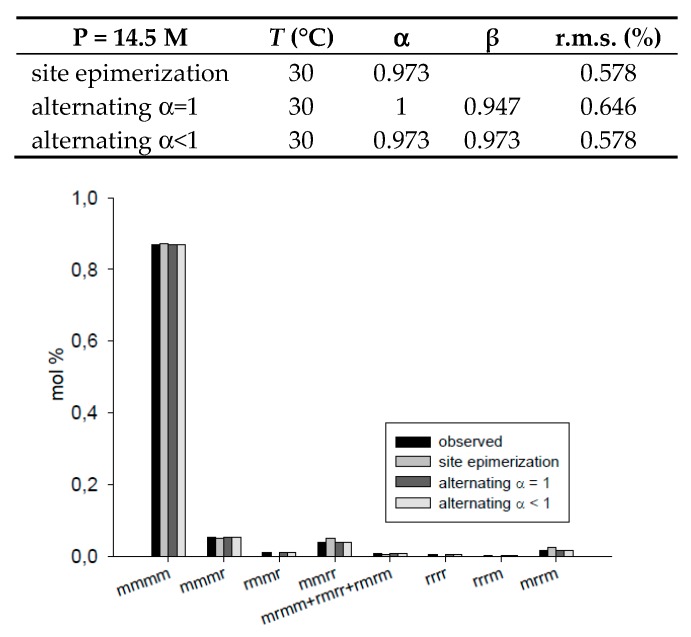

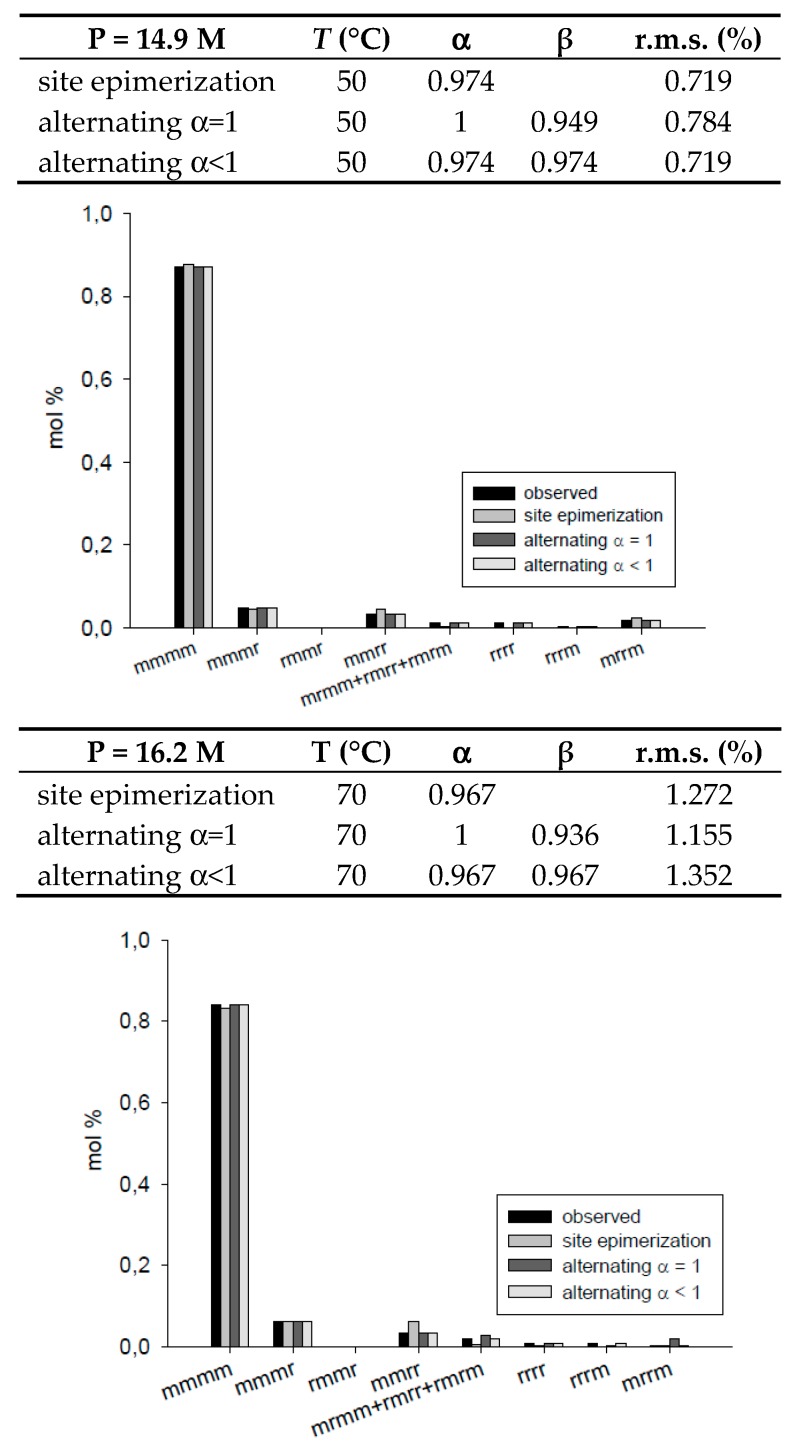
Statistical pentad analysis of a polypropene sample prepared with **3**/MAO at different temperatures.

As far as catalyst **3** is concerned, the addition of a second *t*-butyl at 3 position of the C, that is on the scaffold of catalyst **2** leads to low r.m.s. errors, very similar to those found for catalyst **4** (Table 6 and Figure 6). Apparently, both alternating and epimerization mechanisms are possible. We interpret these data as an indication that, indeed, Cossee’s [16] *alternating mechanism*, that is, the chain migratory insertion, occurs, but the presence of a second *t*-butyl on the same half-space makes the epimerization necessary. This is confirmed by the slight changes in the microstructure with temperature or monomer concentration. Indeed, it is possible to detect an increased isotacticity with the rise in polymerization temperature, at the same propene concentration, and a decrease in isotacticity by increasing the propene concentration (see Table 2) in line with what is expected when the alternating mechanism is operating and the site epimerization mechanism is accessible [20]. The lower activity of catalyst **3** with respect to catalyst **2** is also in line with the need for the polymer chain to back skip (or epimerize) to the original position before the subsequent insertion, that is, almost all the insertions occur on the same site. These results are in line with those found by Tritto et al. [24] in studies in which the statistical model best describing ethylene (E)-norbornene (N) copolymerization with *C*_1_ symmetric catalysts. It was found that norbornene and ethylene are inserted at the same site by a Cossee’s migratory mechanism and a subsequent backskip of the copolymer chain to its original position after every insertion.

**Figure 7:**
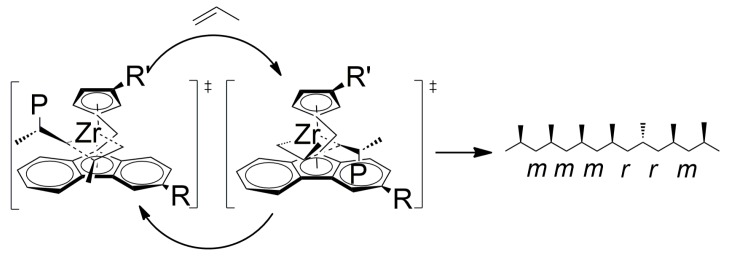
Enantiomorphic site control polymerization mechanism with catalyst **3**: chain migratory mechanism and backskips of the polymer chain to its original position (epimerization) after every insertion.

Thus, even though recent DFT computational studies conclude that it is difficult to assess whether a site epimerization-controlled mechanism takes place in these polymerizations [25], our proposed hypothesis regarding the polymerization mechanism with catalyst **3** is illustrated in Figure 7: the enantiomorphic site control with chain migratory mechanism followed by back-skip of the polymer chain to its original position (site epimerization) after every insertion leads to isotactic polypropene. A similar explanation termed “chain stationary” was proposed by Razavi [26,27] to explain the isotacticity of PP with *C*_1_ symmetric catalysts based on the assumption that the subsequent incoming monomers would “see“ the polymer chain always residing at the same enantio-topic site.

**Figure 8:**
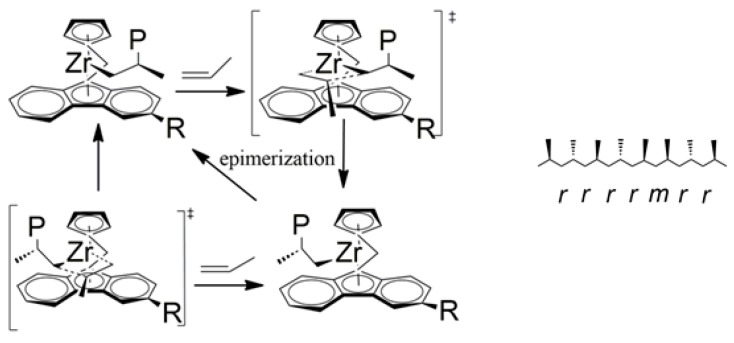
Enantiomorphic site control polymerization mechanism with catalysts **1** and **2**: chain migratory mechanism with accessible backskips of the polymer chain to its original position.

Figure 8 depicts the enantiomorphic site control polymerization mechanism with catalysts **1** and **2** with back-skips of the copolymer chain accessible leading to syndiotactic polypropene with *rrmrr* pentads. This depends on the polymerization temperature and pressure as well as modification of the ligand or the bridge. A high probability of chain backskip and thus a strong competition between site epimerization and chain propagation was observed in E-N copolymerization with *C*_s_-metallocene [28].

### 3.3. ^1^H NMR Analysis

**Figure 9:**
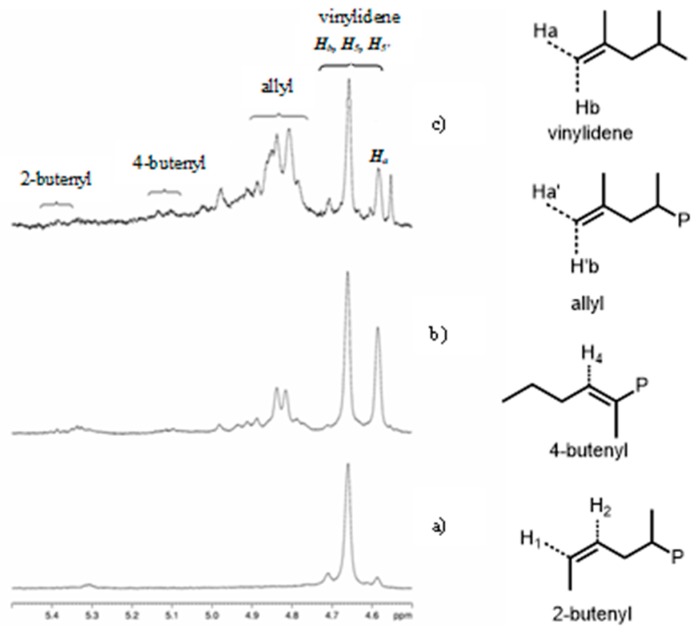
Olefinic region expansion of ^1^H NMR spectra (108.58 MHz, C_2_D_2_Cl_4_, 103 °C) of PP samples prepared at 50 °C by: catalyst **3** (**a**); catalyst **2** at Propene = 13.9 M (**b**); and catalyst **1** (**c**).

The analysis of the integrated signals of the ^1^H NMR spectra of the polymers permits the identification and quantification of the unsaturated terminal end groups. This allows us to determine chain-transfer mechanisms and thus to get information regarding the relationship between catalyst structure and polymer molar masses. The expansions of the olefinic region of ^1^H NMR spectra of polypropene obtained with precatalysts **1**, **2**, and **3**, activated with MAO, are displayed in Figure 9. The main four unsaturated end groups identified in polypropene are: vinylidene, allyl, 2-butenyl and 4-butenyl [29,25] (**H_a_** Singlet 4.59, **H_b_** Singlet 4.66, **H’_a_** Broad Singlet 4.88 ppm, **H’_b_** Doublet 4.94 ppm (*J* = 6.2 Hz), **H^1^** Complex Multiplet 5.72 ppm, **H^2^** Complex Multiplet 5.38 ppm, and **H^4^** Triplet of Multiplets: 5.11 ppm (*J* = 7.0 Hz)).

**Scheme 2:**
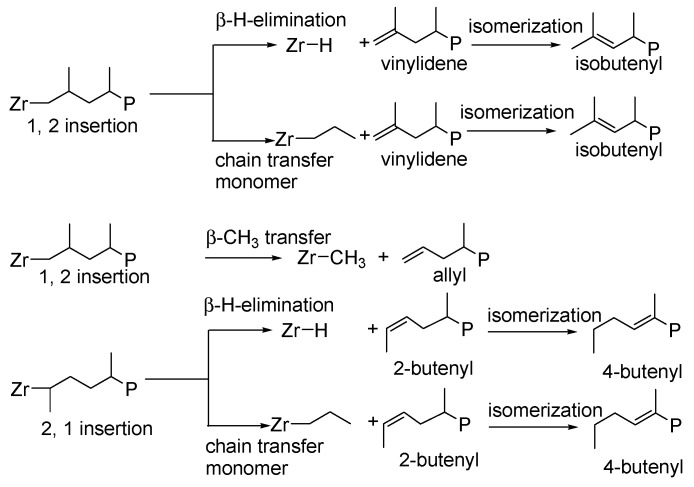
Unsaturated chain terminal group in polypropene.

Scheme 2 summarizes the termination processes of propene polymerization, which are generally recognized as leading to the formation of the unsaturated end-groups displayed in Figure 9.

**Scheme 3:**
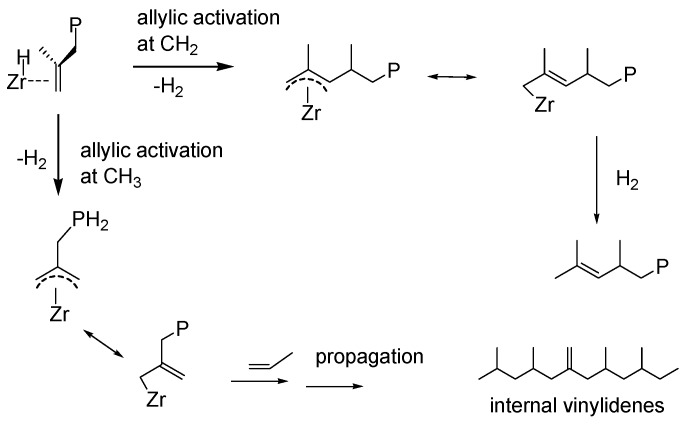
Formation of internal vinylidenes by propene insertion at the η^3^-allyl intermediate

Among the possible chain termination reactions, chain transfer to monomer after a 1, 2 propene insertion is the predominant termination pathway for metallocene based catalysts. The β-H transfer mechanism involves the transfer of a β-H from the polymeryl chain to the β-C of the monomer and leads to vinylidene end groups. Vinylidenes can also arise from β-H elimination, which is a unimolecular process and thus is not influenced by monomer concentration. Chain end groups arising from similar pathways after a last 2, 1 propene insertion are 2-butenyl end and 4-butenyl end groups, which thus are indicative of possible dormant sites [29,30,31,32,33]. Indeed, 4-butenyl end groups are always observed when 2-butenyl end groups are present; they have been proposed to arise from isomerization of 2-butenyl end groups. Coughlin [33] has suggested that this isomerization occurs during the polymerization process and is a reaction catalyzed by the zirconocene hydride complex formed after β-hydride elimination. Theoretically, the two protons of vinylidene groups should have the same intensity, but the areas of the peaks at 4.59 and 4.66 ppm in Figure 9 are not the same. This has been reported by several authors such as Resconi [31] and Kawahara et al. [32] and is due to the different chemical environment of the internal vinylidene (H^5^ and H’^5^), with resonance at 4.68 ppm close to the low field resonance of H_b_ proton. The internal vinylidenes arise from allylic activation, which involves development of H_2_, which can act as chain transfer and lead to the formation of saturated chain end groups (Scheme 3).

**Figure 10:**
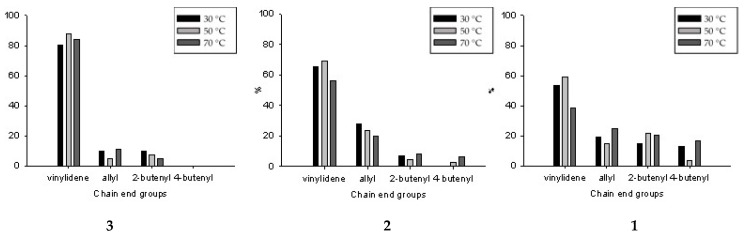
Relative changes of unsaturated end groups observed in PP obtained with **1**, **2**, and **3** catalysts at different temperatures. Vinylidene is the sum of vinylidene, internal vinylidene and isobutenyl end groups.

The relative changes of unsaturated terminal end groups observed in the polymers obtained with **1**, **2** and **3** catalysts at different polymerization temperatures are displayed in Figure 10, where “vinylidene” is the sum of vinylidenes, both internal vinylidene and isobutenyl end groups, since the latter two also arise from vinylidenes. Inspection of Figure 10 reveals that the chain transfer to monomer after a normal 1, 2 insertion is the most probable transfer mechanism in polymerization with metallocene based catalysts. Moreover, the pattern of relative intensities of chain end groups observed is peculiar to each catalyst. The relative amount of typical chain end groups changes slightly with temperature, though a direct relationship is not clear since monomer concentration has an influence too (see below). A first observation is that 2- and 4-butenyl chain end groups are quite low with metallocenes **2** and **3**, which indicates that these catalysts are highly regioselective, while metallocene **1** is less regioselective because of structural modifications induced by the diphenyl on the bridge. It is worth noting that with catalyst **1** all types of chain end groups are possible, vinylidene end groups being only twice the other end groups arising from chain terminations after 2, 1 insertion or by methyl transfer; thus, with catalyst **1,** the relative vinylidene end groups are the lowest, while allyl end groups obtained from β-methyl transfer are the highest.

A comparison between metallocenes **3** and **2** reveals that for polymers produced with metallocene **2** the allyl chain end groups are relevant, thus the presence of only one *t*-butyl substituent makes the transfer of the larger methyl group possible. Similarly, for catalyst **1,** with one *t*-butyl substituent, the β-methyl abstraction is a competitive chain termination mechanism. The changes in allyl group percentages are interesting, indeed, while vinylidenes and butenyl end groups can arise from either a unimolecular or a bimolecular process, allyl end groups can only arise from a unimolecular methyl transfer process. Thus, they should be independent from monomer concentration, conversely; there is a clear relationship between propene concentration in polymerization and allyl end groups; the greater the propene concentration the lower the allyl end groups. The opposite trend is in general evident for vinylidene end groups, that is, those obtained by β-H elimination or transfer to the monomer after a normal 1, 2 insertion. This observation confirms that the transfer to monomer after a normal 1, 2 insertion is the most probable chain transfer in polymerization with metallocene based catalysts.

Such analysis now allows us to observe that there is a correlation between the relative vinilydene end groups of each polymer and the polypropene molar masses found. If we compare the average results of molar masses for each polymer produced with catalysts **1** and **2** (Table 1) with the average vinylidene end groups percentage shown in Figure 10, it is evident that the lower the vinylidene end groups, the higher the molar masses.

Finally, we point out that, in Figure 9, it is clear that the relative intensity between external and internal vinylidenes (see the different peak area of signals at 4.59 and 4.68 ppm) for polymers obtained with the different catalysts is **3** > **1** > **2**, the same order of the pentads arising from epimerization. A mechanism involving dihydrogen/η^3^-allyl complex intermediates was put forward by Resconi to explain epimerization events in *C*_2_-symmetric catalysts [31]. Our results seem to indicate that dihydrogen/η^3^-allyl complex intermediates may contribute to chain epimerization in *C*_1_-symmetric catalysts.

## 4. Conclusions

Propene homopolymers have been synthesized with three *C*_1_-symmetric metallocenes (**1**, **2** and **3**), having *t*-butyl substituents on the fluorenyl moiety or on both moieties, and methylaluminoxane (MAO) at different polymerization temperatures and monomer concentrations. The analysis of polymer microstructure obtained by ^13^C NMR spectroscopy and by statistical analysis suggests that the alternating mechanism, that is, the chain migratory insertion, occurs, but the presence of *t*-butyl on the *C*p renders the site epimerization mechanism competitive. The presence of a second *t*-butyl on the same half-space makes the epimerization necessary. The lower activity of catalyst **3** with respect to catalyst **2** is also in line with the need for the polymer chain to back skip (or epimerize) to the original position before the subsequent insertion; that is, almost all the insertions occur on the same site.

Finally, chain end groups were determined by ^1^H NMR analysis. The changes in allyl groups are interesting, indeed, while vinylidenes and butenyl end groups can arise from either a unimolecular or a bimolecular process, allyl groups arise only from a unimolecular process. Thus, they should be independent of monomer concentration. Conversely, there is a clear relationship between propene concentration in polymerization and allyl end groups: the greater the propene concentration, the lower the allyl end groups. Vinylidene end groups, obtained by β-H elimination or transfer to the monomer after a normal 1, 2 insertion, prevail, indicating that the transfer to monomer after a normal 1, 2 insertion is the most probable chain transfer in polymerization with metallocene based catalysts. Moreover, there is a correlation between the relative vinilydene end groups of each catalyst and the polypropene molar masses found; indeed, the lower the relative vinylidene end groups, the higher the molar masses.

## Figures and Tables

**Table 1:** Propene Homopolymerization in Autoclave: Activity and Molar Masses ^1^.

Entry	Catalyst	*T* (°C)	Propene (mol_P_/V)	Pressure (bar)	Activity ^2^ (Kg_PP_/mol_Zr_·h·P)	*M*_w_^3^ (Kg/mol)	(*M*_w_/*M*_n_)
1	1	30	5.3	4.7	2780	247	2.9
2	1	50	12.0	10.5	951	221	2.1
3	1	70	3.8	6.9	1026	129	2.1
4	2	30	12.5	7.7	1043	141	1.7
5	2	50	10.4	7.2	-	167	1.9
6	2	50	13.3	11.6	721	102	1.9
7	2	50	17.7	12.9	669	108	1.9
8	2	70	12.9	15.4	238	73	1.9
9	3	30	14.5	8.2	337	224	2.1
10	3	50	14.9	12.0	187	228	1.6
11	3	70	16.7	16.6	152	94	1.7

^1^ Zr = 5 μmol; MAO: Al/Zr = 3000/1; Vtoluene = 50 mL. ^2^ Activity in kg_PP_·mol·Zr^−1^·h^−1^·P^−1^. ^3^ Determined by SEC in *o*-C_6_H_4_Cl_2_ with standard PS calibration.

**Table 2:** Microstructure at Pentad Level of the Homopolymers Obtained.

Entry	Catalyst	*T* (°C)	Propene (mol_P_/V)	Pressure (bar)	*rrrr* ^1^ (%)	*mmmm* ^1^ (%)	*rmrr* ^1^ (%)	*rmmr* ^1^ (%)
1	1	30	5.3	4.7	69.8	0.8	10.5	2.1
2	1	50	12.0	10.5	54.1	1.2	17.4	2.9
3	1	70	3.8	6.9	58.9	0.8	15.5	2.6
4	2	30	12.5	7.7	77.4	0.8	5.8	2.1
5	2	50	10.4	7.2	75.8	1.9	7.5	1.8
6	2	50	13.3	11.6	77.6	0.7	6.0	2.0
7	2	50	17.7	12.9	69.4	1.9	9.1	2.4
8	2	70	12.9	15.4	68.0	1.2	8.9	2.6
9	3	30	14.5	8.2	0.56	86.9	0.7	0.9
10	3	50	14.9	12.0	1.25	87.3	1.2	0.0
11	3	70	16.7	16.6	1.01	84.0	1.9	1.0

**^1^** Determined by ^13^C NMR in C_2_D_2_Cl_4_ at 103 °C with HMDS as reference.

**Table 3:** The r.m.s. errors for statistical models for propene polymerizations made with **4**/methylaluminoxane (MAO) and **5**/MAO.

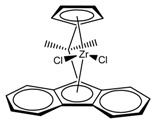		5		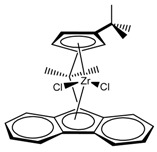		4	
*T* = 50 °C	α	β	r.m.s. (%)	*T* = 40 °C	α	β	r.m.s. (%)
site epimerization	0.631		29.45	site epimerization	0.952		0.687
alternating α = 1	1	0.054	1.66	alternating α = 1	1	0.906	0.886
alternating α < 1	0.972	0.025	1.52	alternating α < 1	0.952	0.952	0.687

**Table 4:** The r.m.s. errors for given statistical models for propene polymerizations made with **2**/MAO.

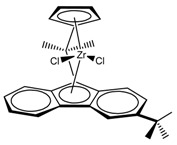			2			
	P (mol/L)	*T* (°C)		α	β	r.m.s. (%)
site epimerization	12.5	30		0.377	/	26.50
	13.3	50		0.379	/	26.57
	12.9	70		0.379	/	24.55
alternating α = 1	12.5	30		1	0.091	2.72
	13.3	50		1	0.090	2.71
	12.9	70		1	0.131	3.88
alternating α < 1	12.5	30		0.952	0.048	2.33
	13.3	50		0.953	0.048	2.32
	12.9	70		0.929	0.073	3.11
site epimerization	13.3	50		0.379	/	26.57
site epimerization	17.7	50		0.376	/	25.07
alternating α = 1	13.3	50		1	0.09	2.71
alternating α = 1	17.7	50		1	0.13	4.03
alternating α < 1	13.3	50		0.953	0.048	2.32
alternating α < 1	17.7	50		0.932	0.048	3.27

**Table 5:** The r.m.s. errors for given statistical models for propene polymerizations made with **1**/MAO.

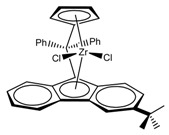		1		
		α	β	r.m.s. (%)
*T* = 30 °C, P = 5.3 M	site epimerization	0.5		23.65
	alternating α = 1	1	0.121	4.30
	alternating α < 1	0.933	0.067	3.57
*T* = 50 °C, P = 12 M	site epimerization	0.5		17.86
	alternating α = 1	1	0.198	6.88
	alternating α < 1	0.881	0.121	4.87
*T* = 70 °C, P = 3.8 M	site epimerization	0.5		20.89
	alternating α = 1	1	0.102	6.33
	alternating α < 1	0.898	0.067	4.75

**Table 6:** The r.m.s. errors for given statistical models for propene polymerizations made with **3**/MAO.

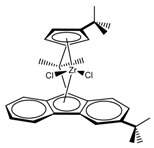		3			
			α	β	r.m.s. (%)
*T* = 30 °C, P = 14.5 M	site epimerization		0.973		0.578
	alternating α = 1		1	0.947	0.646
	alternating α < 1		0.973	0.973	0.578
*T* = 50 °C, P = 14.9 M	site epimerization		0.974		0.719
	alternating α = 1		1	0.949	0.784
	alternating α < 1		0.974	0.974	0.719
*T* = 70 °C, P = 16.2 M	site epimerization		0.967		1.272
	alternating α = 1		1	0.936	1.155
	alternating α < 1		0.967	0.967	1.352
